# Severe Hypovolemic Hyponatremia Following Percutaneous External Biliary Drainage in a Patient With Metastatic Lung Adenocarcinoma: A Case Report

**DOI:** 10.7759/cureus.113269

**Published:** 2026-07-24

**Authors:** Fedonas-Charis Galanis, Christos G Nikolaidis, Andreas D Kyvetos, Georgios Boulmetis, Ioannis Vrettos

**Affiliations:** 1 2nd Department of Internal Medicine, General Oncology Hospital of Kifissia “Agioi Anargyroi”, Athens, GRC; 2 Department of Cardiology, Evangelismos General Hospital, Athens, GRC

**Keywords:** acute kidney injury, biliary drainage, hyponatremia, malignant biliary obstruction, sodium wasting

## Abstract

Hyponatremia is a frequent electrolyte disorder that results from an imbalance between body water and sodium. While hypovolemic hyponatremia has been largely attributed to the loss of sodium either from the gut or the kidneys, sodium depletion caused by external biliary diversion remains a largely uncommon but underacknowledged condition.

We report a case of a 78-year-old man with metastatic adenocarcinoma of the lung who developed severe symptomatic hyponatremia after a percutaneous transhepatic biliary drainage procedure for malignant obstructive jaundice due to metastases to the liver. He complained of generalized weakness, severe fatigue, and nausea. Laboratory analysis showed severe hyponatremia with a serum sodium level of 118 mmol/L, volume depletion, and acute kidney injury due to a prerenal cause. Urinalysis was compatible with extrarenal sodium loss. Further analysis revealed a high sodium concentration in the biliary drainage fluid, plus high daily bile output. These findings strongly suggested biliary sodium loss as the primary mechanism of hyponatremia. The patient was treated with cautious intravenous crystalloid administration and gradual correction of serum sodium levels. The hyponatremia completely resolved, as did the renal function.

This case highlights the importance of close monitoring of fluid and electrolyte balance in patients with external biliary drainage, especially those with high-output drainage, simply due to the fact that the condition can potentially lead to severe life-threatening manifestations such as severe hyponatremia and neurological disturbances.

## Introduction

Hyponatremia is a common electrolyte disorder resulting from an excess of total body water relative to total body sodium [1]. It is defined as a serum sodium concentration below 135 mEq/L [2]. Based on the extracellular fluid volume status, the etiology of hyponatremia can be categorized as hypovolemic, euvolemic, or hypervolemic [3]. Causes of hypovolemic hyponatremia, where the decrease in total body water is less than the decrease in total body sodium, include gastrointestinal fluid losses such as diarrhea or vomiting, diuretic therapy, third-space losses such as pancreatitis, hypoalbuminemia, or small bowel obstruction, cerebral salt-wasting syndrome, salt-wasting nephropathies, mineralocorticoid deficiency, and osmotic diuresis [4]. Among the unusual causes of hypovolemic hyponatremia, percutaneous biliary drainage has been reported [5-7].

In this report, we describe a 78-year-old male patient with symptomatic hyponatremia that occurred five days after the placement of a percutaneous biliary drainage catheter to relieve obstructive jaundice due to liver metastases of lung cancer.

## Case presentation

**Figure 1:**
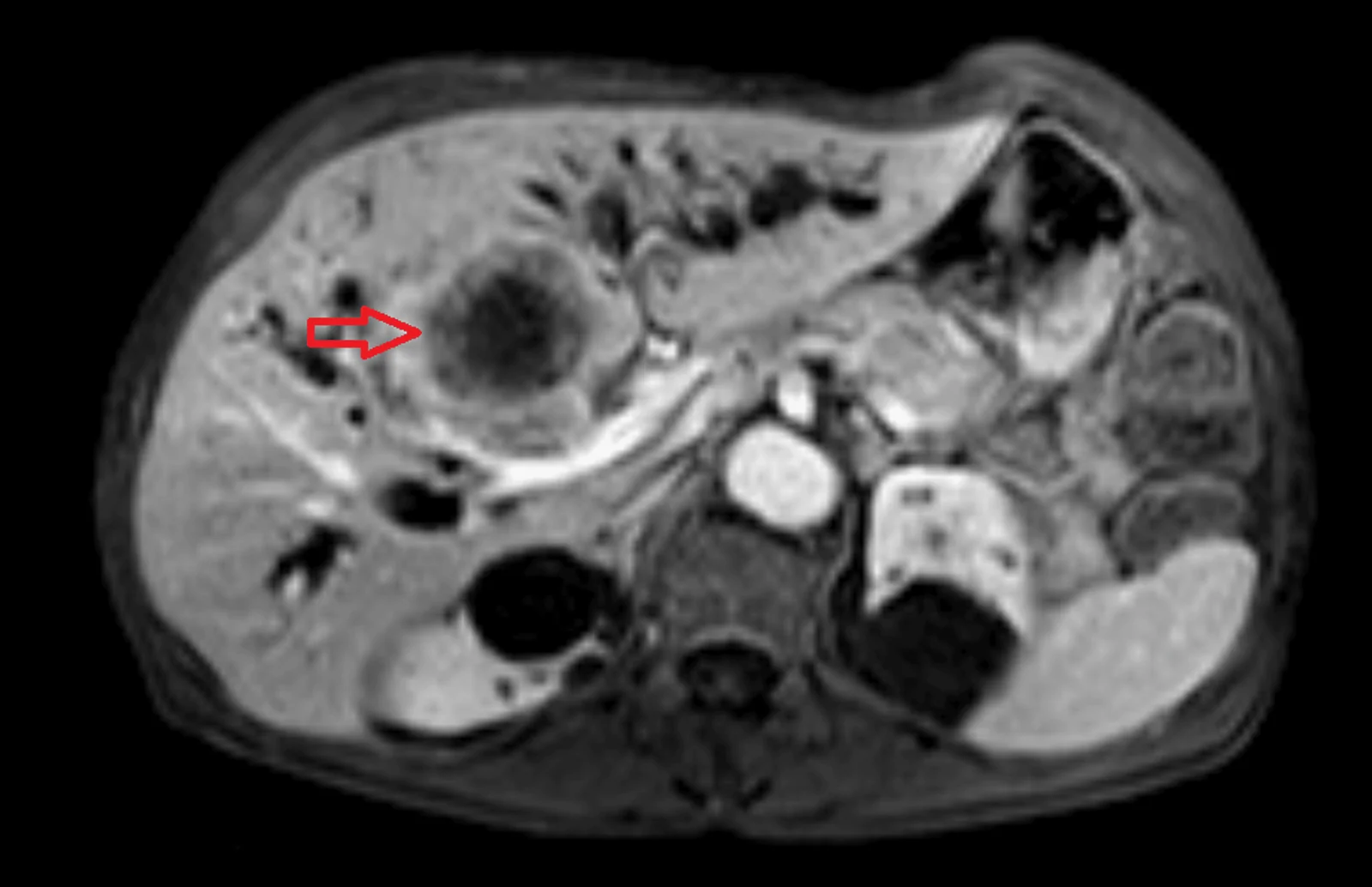
Axial contrast-enhanced T1-weighted MRI image demonstrating a large metastatic lesion in hepatic segment IV (red arrow). MRI: magnetic resonance imaging

**Figure 2:**
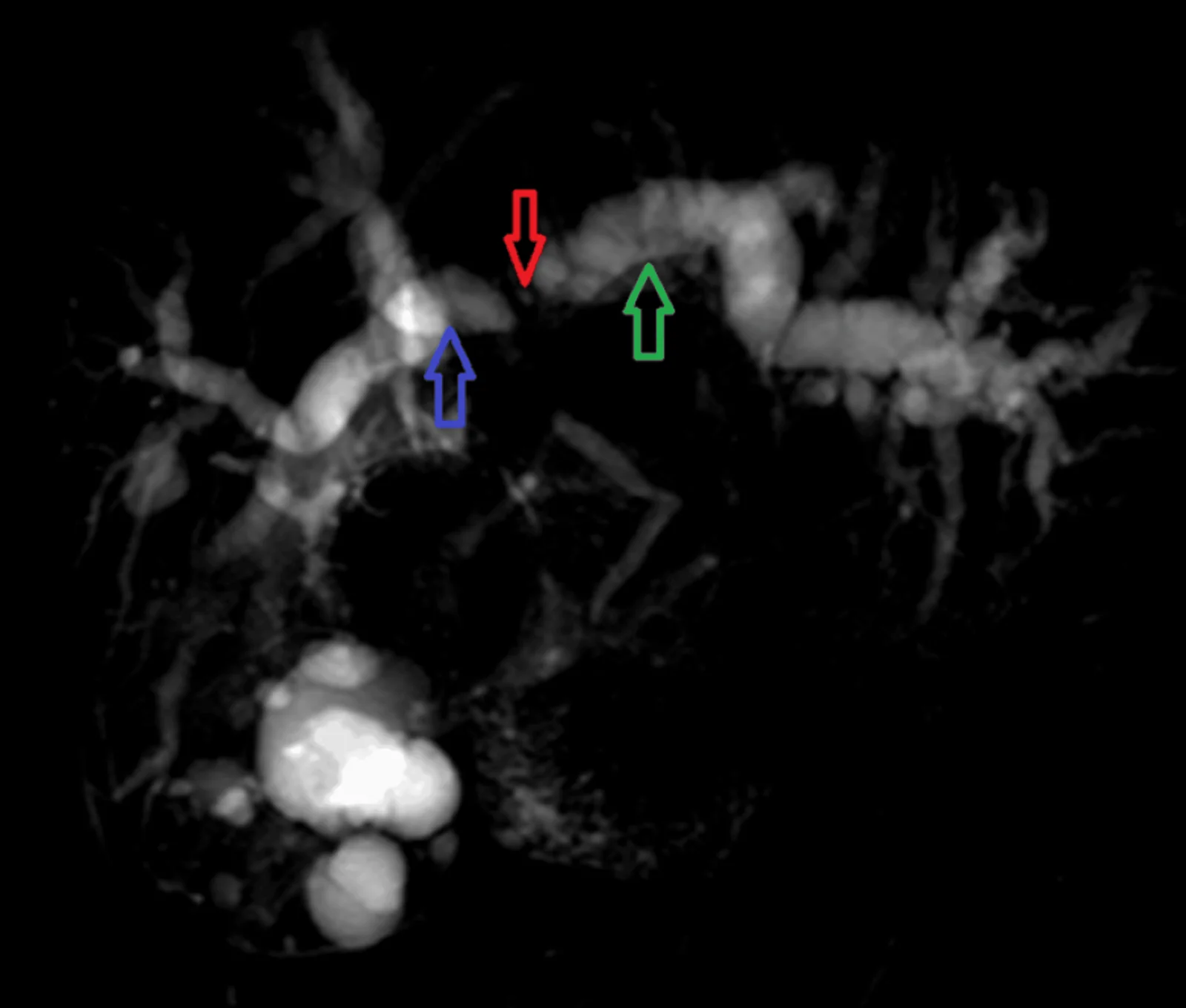
MRCP demonstrating marked dilatation of the right (blue arrow) and left (green arrow) intrahepatic bile ducts secondary to extrinsic compression at the hepatic duct confluence (red arrow), consistent with malignant hilar biliary obstruction. MRCP: magnetic resonance cholangiopancreatography

A 78-year-old male patient presented to the hospital due to pathological findings on a computed tomography (CT) scan performed on an outpatient basis. The imaging findings revealed a space-occupying lesion measuring 4.4 cm in the right lower lobe, with metastatic sites in the liver. His past medical history was unremarkable. Physical examination revealed a palpable liver and diminished breath sounds in the right lower lobe during auscultation. Vital signs showed a blood pressure of 135/86 mmHg and a pulse rate of 68 bpm. The patient underwent a liver biopsy, which confirmed lung adenocarcinoma. During his hospitalization, the patient exhibited elevated levels of direct bilirubin (7.85 mg/dL) and underwent magnetic resonance imaging (MRI) and magnetic resonance cholangiopancreatography (MRCP). MRI and MRCP revealed a metastatic mass in segment IV of the liver, with a diameter of 9.6 cm, causing compressive phenomena on the biliary tree at the confluence of the hepatic ducts with the common hepatic duct, resulting in dilation of the intrahepatic bile ducts (Figures 1-2). The patient underwent percutaneous transhepatic biliary drainage, and bilirubin levels progressively improved. Subsequently, the patient was discharged with instructions for oncological follow-up and management.

After 10 days, the patient presented to the Emergency Department due to general weakness and severe fatigue accompanied by nausea and two episodes of vomiting. On initial examination, vital signs showed a blood pressure of 100/67 mmHg, a pulse rate of 83 bpm, oxygen saturation of 97% (at room air), and a body temperature of 36°C. Physical examination revealed jaundice, a palpable liver with hepatomegaly, poor skin turgor, delayed capillary refill, dry mucous membranes, orthostatic hypotension, and decreased jugular venous pressure. The patient appeared clinically hypovolemic, with no peripheral edema, ascites, or other evidence of third spacing. Laboratory tests revealed hyponatremia (Na: 118 mmol/L) and acute kidney injury (Table 1). The patient did not exhibit any clinical features suggestive of hyponatremic encephalopathy. Additionally, there was no history of diarrhea or diuretic use that could account for the patient's hyponatremia or volume depletion.

**Table 1:** Laboratory investigations.

Blood Tests	Normal Values	Initial Visit	Second Visit	Discharge
Urea (mg/dL)	17-43	27	148	17
Creatinine (mg/dL)	0.66-1.44	0.71	1.29	0.58
K (mmol/L)	3.5-5.1	4.5	5.0	3.3
Na (mmol/L)	136-146	139	118	136
Cl (mmol/L)	101-109	103	81	96
Ca (mg/dL)	8.6-10.6	9.1	8.5	8.8
Glucose (mg/dL)	70-99	68	78	93
Total bilirubin (mg/dL)	0.3-1.2	0.65	3.80	2.10
Direct bilirubin (mg/dL)	<0.2	0.15	2.10	0.97
Aspartate aminotransferase (U/L)	<50	83	46	26
Alanine aminotransferase (U/L)	<50	113	61	18
γ-glutamyl transferase (U/L)	<55	372	507	400
Alkaline phosphatase (U/L)	30-120	393	336	315
Urine Na (mmol/L)	-	-	8	-
Urine creatinine (mg/dL)	-	-	69	-
Triglycerides (mg/dL)	<150	-	311	-
Total protein (g/dL)	6.6-8.3	-	5.4	5.2
Albumin (g/dL)	3.5-5.2	-	2.4	2.7
T3 (ng/mL)	0.28-1.63	-	0.34	-
T4 (μg/dL)	5.85-14.16	-	8.23	-
TSH (μIU/mL)	0.38-5.33	-	2.79	-
White blood cells (K/μL)	4-11	10.72	26.38	24.20
Neutrophils (K/μL)	2.5-7.5	8.90	25.46	22.68
Hematocrit (%)	42-54	36.8	33.4	28.7
Platelets (K/μL)	150-400	459	262	353
Prothrombin time (sec)	10-15	12.6	11.7	15.5
INR	0.85-1.2	1.13	1.57	1.44

The patient was admitted to the Internal Medicine Unit. The acute kidney injury (urea: 148 mg/dL and creatinine: 1.29 mg/dL), with a fractional excretion of sodium (FENa) of 0.1%, was attributed to prerenal causes. Moreover, pseudohyponatremia was excluded based on the normal glucose value and by obtaining a lipid profile and serum protein panel, both of which were within normal limits. His serum osmolality was 265 mOsm/kg, which classified the hyponatremia as hypotonic. Subsequently, because of a low urine sodium concentration (spot urine sodium: 8 mmol/L), hyponatremia was attributed to extrarenal causes. The sodium concentration in bile was 133 mEq/L, and percutaneous drainage of the biliary vessels yielded an average of 680 mL per day. Excluding other causes, percutaneous transhepatic biliary drainage was considered the cause of hyponatremia. The patient received crystalloids, and the next morning, sodium levels were 123 mmol/L. Over the subsequent two days, sodium levels reached 133 mmol/L. Serum sodium was corrected gradually to avoid osmotic demyelination syndrome. Sodium and urea levels were fully restored, and the patient was discharged with a sodium level of 136 mmol/L and a urea level of 17 mg/dL.

## Discussion

Malignant biliary obstruction is common in patients with advanced malignant tumors, leading to poor prognosis and hindering antitumor therapy [8]. In the setting of malignant biliary obstruction due to unresectable malignant tumors, the placement of percutaneous biliary catheters for drainage may be helpful in relieving obstructive symptoms [9]. Although percutaneous transhepatic biliary drainage is a safe and effective way to relieve jaundice caused by advanced inoperable malignant disease [9], procedure-related complications could be observed, such as infectious complications, occlusion or dislocation of drainage, and bile leakage [10]. Moreover, after the placement of a percutaneous biliary catheter in a patient, the importance of fluid and electrolyte replacement has been emphasized in the past [11]. Bile is a physiological aqueous solution produced and secreted by the liver in quantities of approximately 600 mL per day. It consists mainly of bile salts, phospholipids, cholesterol, conjugated bilirubin, electrolytes, and water [12]. The concentration of inorganic solutes (sodium, potassium, calcium, and bicarbonate) in bile is similar to that in plasma and accounts for the bile osmolality of approximately 300 mOsm/kg [13]. Bile has a high sodium concentration, similar to that of plasma, which is essential for bile’s tonicity [14]. After the placement of percutaneous biliary drainage, bile salt reabsorption in the gut, which prevents sodium wasting, is lacking, and a routine daily diet cannot replace external sodium losses in some cases [6].

Indeed, our patient developed dehydration and severe hyponatremia after the percutaneous procedure, which manifested with weakness, fatigue, nausea, and two episodes of vomiting. The patient had a hypovolemic condition, although he did not report diarrhea, sweating, diuretic use, or other causes of hypovolemic hyponatremia. As the urine sodium concentration was less than 20 mmol/L, sodium wasting was considered extrarenal. Initially, gastrointestinal sodium wasting due to vomiting was suspected, but there were only two episodes that stopped soon after. The drainage fluid assessment results supported the occurrence of sodium wasting through biliary drainage. Although the average biliary output of approximately 700 mL/day would not generally be classified as high-output drainage, the measured sodium concentration of 133 mEq/L resulted in daily sodium losses of approximately 93 mEq. This corresponds to approximately 2.1 g of elemental sodium or 5.4 g of sodium chloride, which is close to the maximum daily salt intake recommended by current dietary guidelines. Furthermore, information regarding the patient’s fluid intake, urine output, and biliary drainage volume prior to admission was unavailable. Therefore, it is possible that sodium and fluid losses at home were even greater than those documented during hospitalization, contributing to the development of hypovolemia, acute kidney injury, and severe hyponatremia.

In a previous report, Subasinghe et al. presented a case of hilar cholangiocarcinoma in a 62-year-old man who experienced persistent severe hyponatremia despite salt replacement during external biliary drainage prior to surgery. Sodium levels were restored to normal after nasojejunal refeeding [5].

In another case, a 76-year-old female patient suffering from an inoperable Klatskin tumor presented with kidney dysfunction and hyponatremia due to daily sodium losses of 164 mmol via biliary drainage fluid that exceeded one liter per day. Her sodium levels were restored and remained stable after adding sodium as oral salt supplementation to the patient’s diet [6]. Likewise, Yassine et al. reported a case of a 51-year-old man with an obstructive Klatskin tumor who developed hyponatremia and functional renal failure after high-volume biliary excretion following external biliary drainage. The authors concluded that external biliary drainage can cause serious hydroelectrolytic complications, particularly during high biliary flows, due to the significant loss of water and solutes through the biliary drain. This condition can result in hyponatremia and circulatory insufficiency, which is the cause of functional renal failure [7].

## Conclusions

External biliary drainage should be recognized as a potential cause of clinically significant sodium depletion, hypovolemia, and acute kidney injury. Regular monitoring of serum electrolytes and volume status is particularly important in patients with prolonged or high-volume biliary drainage. Early recognition and appropriate fluid and sodium replacement may prevent severe complications, including symptomatic hyponatremia and renal dysfunction.
